# Pharmacological mechanism of active components in *Polygonatum odoratum* for idiopathic pulmonary fibrosis: a study integrating bioinformatics and experimental validation

**DOI:** 10.3389/fphar.2026.1717994

**Published:** 2026-03-02

**Authors:** Xuedan Cao, Shixuan Kuang, Keyi Jiang, Yaqing He, Fengfu Luo, Yuying Li, Miao Zhang, Dong He, Qun Liang

**Affiliations:** 1 Department of ICU, The First Affiliated Hospital, Heilongjiang University of Chinese Medicine, Harbin, China; 2 School of Basic Medical Sciences, Xiangnan University, Chengzhou, China; 3 School of Basic Medical Sciences, Heilongjiang University of Chinese Medicine, Harbin, China

**Keywords:** bioinformatics, idiopathic pulmonary fibrosis, n-coumaroyltyramine, pharmacological mechanism, *Polygonatum odoratum*

## Abstract

**Introduction:** Idiopathic Pulmonary Fibrosis (IPF) is a chronic, progressive, and often fatal interstitial lung disease characterized by persistent alveolar injury, inflammation, and extracellular matrix remodeling, ultimately leading to respiratory failure. Despite ongoing research, current therapeutic options for IPF remain limited, underscoring the urgent need for novel treatment strategies.

**Methods:** In this study, we investigated the pharmacological mechanisms of *Polygonatum odoratum* (PO) in treating IPF. We employed Weighted Gene Coexpression Network Analysis (WGCNA) and network pharmacology to identify potential therapeutic targets. Molecular docking and molecular dynamics simulations were conducted to evaluate the binding affinities and structural stability of key bioactive compounds. Subsequently, experimental validation was performed using a cellular model of bleomycin-induced pulmonary fibrosis.

**Results:** Network analysis identified central carbon metabolism and the PI3K-Akt signaling pathway as key associated pathways. Molecular docking demonstrated that bioactive compounds of PO (including MOL010412 and MOL000332) exhibited strong binding affinities to core targets such as EGFR, BCL2, MTOR, HIF1A, and GSK3B. Experimental results confirmed that MOL000332 (n-coumaroyltyramine) significantly mitigated pulmonary fibrosis by suppressing the protein expression levels of EGFR, HIF1A, and GSK3B.

**Discussion:** These findings suggested that PO exerted its therapeutic effects through the modulation of multiple targets and pathways, positioning it as a promising candidate for IPF treatment. This study provided a robust scientific foundation for further exploration and development of PO-based therapies for IPF.

## Intoduction

Pulmonary fibrosis (PF), marked by diffuse alveolitis and disruption of the alveolar architecture, is an interstitial lung disease defined by abnormal fibroblast proliferation and excessive deposition of connective tissue (Richeldi et al., 2003). Chronic inflammation and progressive fibrosis of the pulmonary interstitium inevitably impaired gas exchange and lead to dyspnea, resulting in a marked decline in quality of life (Kim et al., 2015; Raghu et al., 2022). The progressive deterioration of PF to advanced stage ultimately resulted in respiratory failure, posing a serious threat to patient survival (Koudstaal et al., 2023). PF, being a chronic progressive disease, represents the end-stage outcome of various interstitial lung diseases (ILDs) and has increasingly been recognized as a major global healthcare challenge (Cheng et al., 2022). Multiple environmental exposures—including asbestos, silica, and fumes—have been associated with oxidative stress, mitochondrial damage, and mitophagy, particularly in PM2.5-induced PF (Gao et al., 2020; Xu et al., 2021). In addition, several viral pathogens, such as Human Immunodeficiency Virus (HIV), avian influenza virus and cytomegalovirus (CMV), have been shown to induce pulmonary fibrosis through viral infection-mediated lung injury and persistent inflammatory infiltration (Sheng et al., 2020; Huang and Tang, 2021). Autoimmune diseases such as sjögren’s syndrome (SS) and rheumatoid arthritis (RA), characterized by dysregulated immunity, could further accelerate inflammation and fibrotic remodeling during PF progression (Shi et al., 2020; Wang et al., 2022b). PF can be broadly classified into two major categories: secondary PF with a defined etiology, and idiopathic pulmonary fibrosis (IPF) for which no causative factor has been identified (Zhao et al., 2023). Among these, IPF is the most common form of ILD and is defined as a relentlessly progressive and typically fatal disease. Once diagnosed, IPF confers a median survival of approximately 3–5 years and progresses inexorably to respiratory failure (Hao et al., 2015). Increasing research has established that epigenetic and genetic abnormalities, together with environmental exposures and viral infections, contributed to the onset and progression of IPF. Preliminary analysis of IPF whole-genome sequencing analyses identified pathogenic variants in genes such as RTEL1 and TERT, which significantly elevate disease risk (Peljto et al., 2023). In addition, hypermethylation of the CD90/Thy-1 promoter has been shown to suppress Thy-1 expression, thereby promoting fibroblast–myofibroblast transition and exacerbating IPF progression (Sanders et al., 2008). A meta-analysis of 22 studies from 12 geographic regions estimated the global incidence of IPF at 1–13 cases per 100,000 persons and a prevalence of 3–45 cases per 100,000 persons (Maher et al., 2021). With population aging, increased air pollution, and other high-risk exposures, the global burden of IPF has been projected to continue rising (Podolanczuk et al., 2023). Notably, the association between IPF and lung cancer has drawn increasing clinical attention. Clinical anatomical findings confirmed that fibrosis in IPF typically involved the peripheral lung and lower lobes—regions that also represented common sites of tumor development (Antoniou et al., 2015). Collectively, IPF was a rare, irreversible, and progressive fibrotic lung disease with a substantial risk of malignant transformation, posing a significant threat to both health and quality of life.

Clinical trials for the treatment of IPF are ongoing; however, available therapeutic options have remained limited. Although pirfenidone and nintedanib, both approved by the U.S. FDA, could slow the progression of fibrosis, recent studies have reported that these immunosuppressive drugs may exert immunosuppressive effects and induce hepatic and renal toxicity (George et al., 2020). Given these limitations, the development of effective and safer therapeutic strategies for IPF has continued to represent a major challenge in pulmonary medicine. Bioactive compounds derived from medicinal plants have demonstrated promising pharmacological activities and have increasingly become a focus of antifibrotic drug discovery. On this basis, numerous studies have explored the potential of natural compounds in PF therapy and have yielded encouraging preliminary findings. For instance, astragaloside IV (AST), derived from *Astragalus membranaceus*, significantly alleviated PF in COPD by inhibiting the RAS/RAF/FoxO signaling pathway (Zhang et al., 2023a). Atractylodinol (ATD), a representative active component of *Atractylodes lancea*, was found to impede the progression of PF by targeting VIM and downregulating the TGF-β/Smad signaling pathway (Hao et al., 2023). Additionally, triptolide has shown potential in ameliorating PF by suppressing pathological extracellular matrix remodeling through modulation of the MMPs/LOX/integrin signaling axis (Lin et al., 2023). Therefore, identifying additional natural plant–derived bioactive compounds with comparable or superior efficacy and reduced toxicity may offer an effective approach to improving clinical outcomes in IPF. In TCM theory, the clinical manifestations of idiopathic pulmonary fibrosis (IPF)—including progressive dyspnea, dry cough, and fatigue—are categorized under “Fei Wei” (“lung wilting”). This term first appeared in <Jin Gui Yao Lue>, and its core pathogenesis is characterized by depletion of lung qi and yin, impaired distribution of body fluids, and loss of nourishment to pulmonary tissues, resulting in atrophy and dysfunction. Over time, chronic illness affects the collaterals and leads to phlegm–blood stasis obstruction, ultimately causing substantive structural damage to the lungs. These descriptions are highly consistent with the modern pathological understanding of IPF, namely, persistent alveolar epithelial injury, aberrant repair, excessive extracellular matrix deposition, and consequent lung architectural remodeling and functional decline.

The *Polygonatum odoratum* (PO), also known as “Yu Zhu” was highly valued in China and Korea for its edible and medicinal properties (Zhao and Li, 2015; Zhou et al., 2015). PO, as an herbal medicine, boasts a time-honored history rooted deeply in traditional Chinese medicine. According to the esteemed ancient text, <Shen Nong Ben Cao Jing>, PO was described as its neutral nature and sweet taste, aligning with the meridians of the lungs and stomach. It was revered for its efficacy in stimulating the production of bodily fluids to quench thirst, nourishing the yin to maintain harmony and rejuvenation, as well as moistening and alleviating dryness, thereby exhibiting a comprehensive range of therapeutic benefits. The Shashen Maidong Decoction, a classic formula that includes PO, Adenophora stricta, and Ophiopogon japonicus, was first recorded in <Systematic Differentiation of Warm Diseases> (Wu Jutong, Qing Dynasty) and remained a foundational prescription for treating dryness-induced lung-stomach injury. Modern clinical studies shown that this formula could effectively improve lung function in patients with chronic bronchitis (Feng, 2020) and enhance therapeutic efficacy in acute exacerbations of chronic obstructive pulmonary disease when used as an adjuvant treatment (Chen et al., 2021). Furthermore, in patients with cough variant asthma attributed to lung yin deficiency, a modified Shashen Maidong Decoction combined with conventional Western medicine yielded significantly better outcomes than Western medicine alone (Wang et al., 2015). These clinical observations provided a practical foundation for further pharmacological investigation into the multi-target mechanisms of PO. What’s more, modern pharmacological research also confirmed that the active components or extracts of PO exerted a broad spectrum of therapeutic effects. PO polysaccharide exhibited a protective effect against LPS-induced lung inflammatory injury, which appeared to be associated with its potential to reduce harmful bacteria and repair the intestinal barrier (Liu et al., 2023). The methanol extract of PO improved ulcerative colitis by modulating the gut microbiota and attenuating inflammation (Ye et al., 2022). Additionally, the rhizome extract of PO, which included polysaccharides, homoisoflavonoids, and saponins, ameliorated gastric pathological damage, possibly through suppressing oxidative stress (Mariod et al., 2023). Despite these advances, evidence regarding the therapeutic efficacy of PO and its active constituents in the treatment of IPF remained scarce.

During the initiation and progression of IPF, primary lung injury (e.g., infection or toxic particle exposure) triggers a sustained inflammatory response (Hosseini et al., 2021; Wynn and Ramalingam, 2012). This process is accompanied by marked oxidative stress, which further exacerbates alveolar epithelial cell damage and induces fibroblast proliferation and activation, thereby driving the fibrotic cascade (Bezerra et al., 2023; Tsukui et al., 2024). Moreover, recent studies have revealed the key role of the bidirectional “gut–lung axis” in pulmonary disorders (Dang and Marsland, 2019). This axis has been shown to participate in the regulation of asthma and acute lung injury (Arrieta et al., 2015; Kapur et al., 2018), and also plays an important role in pulmonary fibrosis (Wu et al., 2022). Evidence has shown that mouse models of pulmonary fibrosis display significant gut microbiota dysbiosis and altered metabolomic profiles (Gong et al., 2021), whereas modulation of gut microbial ecology through fecal microbiota transplantation markedly improves fibrotic outcomes (Gurczynski et al., 2023). In this study, we sought to investigate the potential pharmacological mechanisms by which PO alleviated IPF through bioinformatics analysis and *in vitro* experimental validation. These findings may provide a biological basis for elucidating the protective effects of PO against IPF and support its further development as a clinically promising candidate for IPF therapy.

## Materials and methods

### Data acquisition and preprocessing

Gene expression analysis data related to IPF, GSE53845, was downloaded from the GEO database. This dataset comprised 40 IPF samples and 8 normal control lung tissue samples. After excluding 11 IPF lung biopsy samples, 8 normal samples and 29 IPF lung transplant samples were retained for analysis. The retained data were standardized and processed for clustering analysis using the WGCNA package. Additionally, corresponding clinical information for the retained data was also obtained from the GEO database for subsequent analyses.

### Weighted gene Co-expression network analysis (WGCNA)

WGCNA was employed to identify gene co-expression modules associated with IPF. Pearson correlation coefficients were first calculated between all gene pairs to generate a similarity matrix, which was converted into an adjacency matrix using soft-thresholding power. Pearson correlation coefficients were first calculated between all gene pairs to generate a similarity matrix, which was converted into an adjacency matrix using an appropriate soft-thresholding power. Based on this, gene modules were identified. Modules with a module eigengene (ME) correlation coefficient greater than 0.5 with clinical traits were considered significant. Module membership (MM) and gene significance (GS) were used to further characterize module–trait relevance, and Modules with a high correlation to IPF progression (similarity greater than 0.5, *p*-value less than 0.05) were identified as significant modules. The genes from these significant modules were then exported for subsequent analysis. To assess module stability, a global co-expression network was constructed using all samples. Module preservation was then evaluated with the modulePreservation function in the WGCNA package, treating the merged network as the reference and the IPF and control subgroups as test networks. A permutation test (nPermutations = 30) generated Z-summary statistics, which quantified the preservation of module structure across subgroups.

### Collection of potential targets of PO

The main chemical components of PO were retrieved from the Traditional Chinese Medicine Systems Pharmacology Database and Analysis Platform (TCMSP, https://www.tcmsp-e.com/#/home). The active components of PO were then identified based on the recommended screening criteria (Oral Bioavailability, OB ≥ 30%, Drug-Likeness, DL ≥ 0.18). Subsequently, potential targets of these active components were predicted using Swiss Target Prediction (http://swisstargetprediction.ch/) and SEA (Similarity Ensemble Approach, https://sea.bkslab.org/) databases. After integrating the results and removing duplicate entries, the potential targets of PO were obtained.

### Identification of common targets between PO and IPF-related disease targets

To explore the correlation between the potential targets of PO’s active components and IPF -associated genes, the key module genes identified by WGCNA were intersected with the potential targets of PO using the Venny 2.1.0 online tool (https://bioinfogp.cnb.csic.es/tools/venny/index.html). A Venn diagram was subsequently generated to visualize the common targets derived from this intersection.

### Construction of protein-protein interaction (PPI) network

The STRING database (https://string-db.org/) was utilized to explore known and predicted protein-protein interaction (PPI). The common targets between PO and IPF were imported into the STRING database, with the organism set to “*Homo sapiens*” and default settings applied. Isolated proteins were excluded to construct the PPI network of the shared targets. The resulting PPI network was then imported into Cytoscape 3.9.1 software, where a network diagram was generated based on node degree to further identify the core targets of PO in the treatment of IPF. Additionally, using the identified active components of PO, an “active component-core target” network for PO’s treatment of IPF was also constructed in Cytoscape 3.9.1 software.

### GO and KEGG enrichment analysis

The core targets of PO in the treatment of IPF were submitted to the DAVID database for Gene Ontology (GO) and Kyoto Encyclopedia of Genes and Genomes (KEGG) pathway enrichment analyses. These analyses were performed to elucidate the involvement of the common targets in biological processes (BP), molecular functions (MF), cellular components (CC), and relevant signaling pathways. The results of the GO and KEGG enrichment analyses were subsequently visualized.

### Molecular docking

Regarding protein and ligand preparation, the protein structures were retrieved from RCSB PDB and processed in Discovery Studio 2019, including removal of crystallographic water and co-crystallized ligands, hydrogen addition, charge completion, and repair of missing side chains/amino acids. The optimized proteins were saved in PDB format. Small-molecule ligands were retrieved from PubChem and subjected to energy minimization under MMFF94 in Discovery Studio before being saved as PDB structures. AutoDockTools 1.5.6 was then applied to generate PDBQT files for both receptors and ligands, including hydrogen addition, charge assignment, and torsion definition. Docking was performed using AutoDock Vina 1.2.6, and visualization was carried out in PyMOL 3.1 and Discovery Studio 2019.

### Molecular dynamics simulation

To further evaluate the dynamic stability of the protein–ligand complexes, molecular dynamics (MD) simulations were performed using GROMACS 2025 installed on a local Linux workstation. The docked complexes of EGFR, HIF1A, and GSK3B with n-coumaroyltyramine were used as the initial structures. Protein topologies were generated using the AMBER14SB force field, while ligand parameters were constructed using the GAFF2 force field with appropriately assigned atom types. The protein and ligand topology files were subsequently merged to ensure complete compatibility in atom definitions. Each complex was placed in a cubic simulation box with periodic boundary conditions (PBC), maintaining a minimum distance of 1.2 nm between any solute atom and the box boundary. The systems were solvated using the TIP3P explicit water model, and Na^+^/Cl^−^ counterions were added to neutralize the total charge and adjust the ionic strength to 0.15 M, mimicking physiological conditions. Energy minimization was performed using the steepest-descent algorithm until the maximum force reached the predefined convergence threshold. The minimized systems were then equilibrated under NVT (constant number of particles, volume, and temperature) and NPT (constant number of particles, pressure, and temperature) ensembles for 2 ns, using a coupling constant of 0.1 ps. Production MD simulations were subsequently conducted for 100 ns at a constant temperature of 310 K (regulated using the V-rescale thermostat) and a pressure of 1 bar (controlled using the Parrinello–Rahman barostat). The integration time step was set to 2 fs, and system coordinates were saved every 1000 steps for downstream analyses. Trajectory analyses included the calculation of root-mean-square deviation (RMSD), root-mean-square fluctuation (RMSF), radius of gyration (Rg), solvent-accessible surface area (SASA), and intermolecular hydrogen bond counts using built-in GROMACS tools. These parameters were used to evaluate the conformational stability and interaction persistence of each complex throughout the simulation.

### Cell culture and seeding

BEAS-2B cell (iCell Bioscience Inc., Shanghai, China) were cultured in Dulbecco’s Modified Eagle Medium (DMEM; Gibco, United States) supplemented with 10% fetal bovine serum (FBS; Gibco, United States) and 1% penicillin-streptomycin (Gibco, United States) at 37 °C in a humidified atmosphere containing 5% CO_2_. Upon reaching approximately 90% confluence, cells were detached using 0.25% trypsin-EDTA (Gibco, United States) and subsequently seeded into 96-well plates at a density of 5 × 10^3^ cells per well, with five replicates prepared for each experimental group.

### Cell viability assay (CCK-8)

Following overnight incubation to ensure proper cell adhesion, the cultured cells were exposed to a concentration gradient of n-coumaroyltyramine (CAS: 36417-86-4, Sangon Biotech., Shanghai, China) (0, 1, 10, 50, 100, and 200 μg/mL) for 48 h. N-Coumaroyltyramine was dissolved in dimethyl sulfoxide (DMSO) and diluted with culture medium to achieve the desired concentrations, with the final DMSO concentration kept at 0.05% (v/v) in all solutions. Cell viability was assessed using the Cell Counting Kit-8 (CCK-8; Sangon Biotech., Shanghai, China) according to the manufacturer’s instructions. Briefly, 10 µL of CCK-8 reagent was added to each well, followed by incubation at 37 °C with 5% CO_2_ for 2 h. The optical density (OD) was then measured at 450 nm using a microplate reader (BioTek, United States) to quantify the effects of n-coumaroyltyramine on cell viability and proliferation.

### Bleomycin-induced IPF in BEAS-2B cells and N-Coumaroyltyramine treatment

Cell culture and seeding were performed as described above. BEAS-2B cells were treated with 10 μg/mL bleomycin (Sigma-Aldrich, United States) for 48 h to induce IPF. After bleomycin exposure, cells were gently washed twice with phosphate-buffered saline (PBS; Gibco, United States) and subsequently treated with 50 μg/mL n-coumaroyltyramine for 48 h. Cellular morphological changes in different groups were systematically observed and documented using an inverted phase-contrast microscope (Nikon, Japan) equipped with a digital imaging system. All observations and imaging were conducted at ×100 magnification.

### Quantitative real-time PCR analysis

Total RNA was extracted using the Total RNA Kit (Takara Bio, Japan) and quantified with a Nanodrop 2000C spectrophotometer (Thermo Fisher Scientific, United States). cDNA was synthesized from 1 μg RNA using PrimeScript RT Master Mix (Takara Bio, Japan). qPCR was performed with SYBR Premix Ex Taq II (Takara Bio, Japan) on a QuantStudio 6 Flex system (Applied Biosystems, United States) under the following conditions: 95 °C for 30 s, followed by 40 cycles of 95 °C for 5 s and 60 °C for 30 s. Gene expression was normalized to β-actin using the 2^−ΔΔCT^ method. Primer sequences were as follows: *HIF1α*: F 5′-GTG​TAC​CCT​AAC​TAG​CCG-3′, R 5′-ACA​AAT​CAG​CAC​CAG​C-3'; *EGFR*: F 5′-CAT​CTC​CGA​AGC​CAA​CA-3′, R 5′-CGA​CGG​TCC​TCA​AAG​TAG-3'; *GSK3β*: F 5′-CAA​CTG​CCC​GAC​TAA​CAC-3′, R 5′-GAG​GAG​GAA​TAA​GGA​TGG-3'; *β-actin*: F 5′-TCA​GGG​TGA​GGA​TGC​CTC​TC-3′, R 5′-CTC​GTC​GTC​GAC​AAC​GGC​T-3′.

### Protein extraction, Western blotting, and antibody detection

Cells were washed with PBS and lysed on ice in RIPA buffer freshly supplemented with a 1× protease and phosphatase inhibitor cocktail (Halt™ Protease and Phosphatase Inhibitor Cocktail, EDTA-free; Thermo Fisher Scientific, Cat. No. 78443). Lysates were incubated on ice for 10 min and centrifuged at 16,000 × g for 15 min at 4 °C. Protein concentrations were determined using the BCA assay (Thermo Fisher Scientific, Cat. No. 23225), and all samples were normalized to 2 μg/μL to ensure equal protein loading. Subsequently, 20 μg of total protein per lane was separated by 10% SDS-PAGE and transferred to PVDF membranes. A protein molecular weight marker (Servicebio, G2508) was used as a reference. Membranes were blocked with 5% non-fat milk in TBST and incubated with the following primary antibodies: HIF1α: Anti-HIF1α (Proteintech, 66730-1-Ig); EGFR: Anti-EGFR (Proteintech, 66455-1-Ig); Phospho-EGFR: Anti-Phospho-EGFR (Tyr 1173) (Sigma-Aldrich, MABS829); GSK3B: Anti-GSK3B (Proteintech, 67329-1-Ig); Phospho-GSK3B (Ser9): Anti-Phospho-GSK3B (Ser9) (Proteintech, 67558-1-Ig). Notably, GAPDH (approx. 36-37 kDa; Anti-GAPDH: Proteintech, 60004-1-Ig) was utilized as the loading control instead of β-actin (approx. 42 kDa). This selection was intended to ensure sufficient electrophoretic separation and prevent potential signal overlap between β-actin and the target proteins (GSK3B and p-GSKB, approx. 46 kDa), given their proximity in molecular weight. After washing with TBST, membranes were incubated with HRP-conjugated secondary antibodies (1:5000) for 1 h at room temperature. Protein bands were visualized by enhanced chemiluminescence and detected using an automated ECL imaging system (Shanghai Tanon 5200 Multi). All Western blot results were derived from three independent biological replicates, each representing a fully independent experiment from cell thawing and chemical treatment to protein extraction and immunoblotting.

### Statistical analysis

All data were expressed as mean ± standard deviation and analyzed using GraphPad Prism 10.4.1. Data normality was assessed by the Shapiro–Wilk test, and homogeneity of variance by Levene’s test. Multiple-group differences were examined using one-way ANOVA, followed by Bonferroni-corrected *post hoc* tests when the overall effect was significant (*p* < 0.05). Comparisons were considered statistically significant only when the corrected P-value was below the adjusted threshold.

## Results

### Weighted gene Co-expression network analysis

**FIGURE 1:**
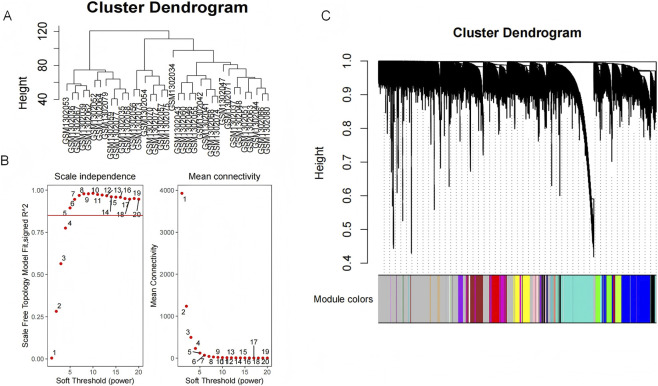
Construction of co-expression module. **(A)** Clustering analysis to detect abnormal sample data. **(B)** Soft-thresholding power analysis to obtain the scale-free fitting index for network topology. **(C)** Hierarchical clustering analysis to detect co-expression clusters with corresponding color assignments.

Weighted Gene Co-Expression Network Analysis (WGCNA) is an effective method for identifying gene modules composed of highly correlated genes and linking these modules with disease phenotypes to determine core genes highly related to the disease state. In this study, the GSE3845 dataset, comprising 29 IPF samples (from lung transplant patients) and 8 normal samples (from normal lung tissue of IPF patients), was utilized for WGCNA analysis. Sample clustering analysis for quality control was shown in Figure 1A. To construct a gene co-expression network approximating a scale-free topology, the soft-thresholding power β was set to 8 (*R* ^2^ = 0.98) (Figures 1B,C), which enhanced the significance and stability of gene co-expression in the network. Subsequent average linkage hierarchical clustering analysis identified 35 significant co-expression modules, with the gray module representing genes that could not be assigned to other significant modules.

### Gene module identification

**FIGURE 2:**
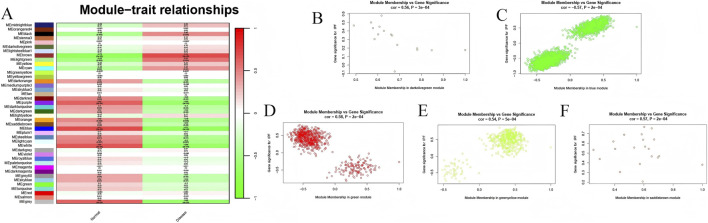
Identification of Hub Genes within Modular Characteristics. **(A)** Correlation between modules and clinical features. **(B–F)** Scatter plots illustrating the relationship between module membership (MM) and gene significance (GS) for the darkolivegreen, blue, green, greenyellow and saddlebrown modules.

Identifying gene modules highly associated with the progression of IPF is a critical approach to uncovering core regulatory targets. Gene modules closely associated with IPF progression were identified by WGCNA. Modules with a correlation coefficient >0.5 and p < 0.05 with clinical traits were considered significant. The results revealed that the darkolivegreen module (R = 0.56, *p* = 0.0003), blue module (R = −0.57, *p* = 0.0002), green module (R = 0.58, *p* = 0.0002), greenyellow module (R = 0.54, *p* = 0.0005), and saddlebrown module (R = 0.57, *p* = 0.0002) exhibited strong correlation with IPF (Figure 2A). Additionally, scatter plots of Module Membership (MM) versus Gene Significance (GS) for the IPF clinical samples confirmed the significant correlation between the selected gene modules and development of IPF (Figures 2B–F). Module preservation analysis demonstrated that the blue, green, and greenyellow modules were highly preserved (Z-summary >10) across IPF and control subgroups, whereas the darkolivegreen and saddlebrown modules were moderately preserved (Z-summary, 2–10), indicating robust co-expression patterns across sample groups (Supplementary Figure S1). Consequently, these 5 modules, comprising a total of 3541 genes, were selected for further investigation.

### Collection of potential targets for PO in the treatment of IPF

**FIGURE 3:**
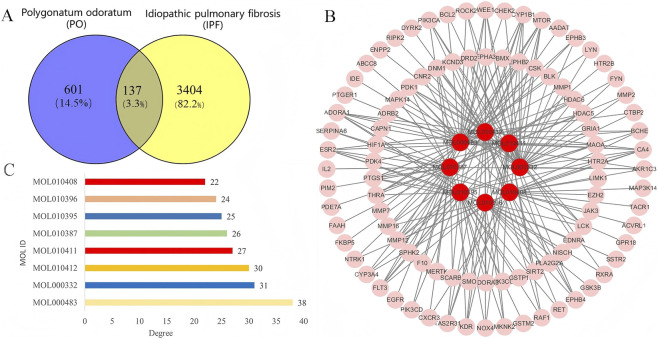
The potential pharmacological targets of Polygonatum odoratum (PO) in the treatment of idiopathic pulmonary fibrosis. **(A) **Venn diagram of the intersection genes of IPF-related modules and the potential targets of PO **(B)** the drug-target interactions of PO active components against IPF-related targets. **(C)** Statistical analysis of PO active components against predicted intersecting targets.

The bioactive chemical components of PO were collected from the Traditional Chinese Medicine Systems Pharmacology (TCMSP) database, resulting in a total of 62 chemical components (Supplementary Data 2). Based on the screening criteria of oral bioavailability (OB ≥ 30%) and drug-likeness (DL ≥ 0.18), 8 components with potential pharmacological activity were selected (Table 1). The potential therapeutic targets for these eight chemical components were subsequently identified using the Swiss Target Prediction and SEA databases. After merging the results and removing duplicates, 738 potential therapeutic targets of PO were obtained (Supplementary Data 3). The intersection between the core genes of IPF-related modules and the potential targets of PO was determined using the Venny 2.1 online tool, resulting in 137 intersection targets (Figure 3A, Supplementary Data 4). These intersection targets were then used to construct a protein-protein interaction (PPI) network, and further analysis in Cytoscape 3.8.2 software delineated the interactions between PO’s active components and IPF-related targets, as shown in Figure 3B. Statistical analysis indicated that PO’s 8 active components could potentially interact with the majority of predicted core targets (Figure 3C), suggesting their contribution to the therapeutic effects of PO.

**TABLE 1:** Active compounds of *Polygonatum odoratum* (PO).

Mol ID	Molecule name	MW	OB (%)	DL	Chemical formula
MOL010387	3-o-beta-d-glucopyranosyl-(1-2)-[beta-d-xylopyranosyl-(1-3)]-beta-d-glucopyranosyl-(1-4)-galactopyranosyl-25(S)-spirost-5 (6)-en-3beta,14alpho-diol_qt	430.69	104.58	0.79	[C@@]12(C(=CC [C@@H]3 [C@@H]1CC [C@]1 ([C@@]3(C[C@H]3 [C@@H]1 [C@@H]([C@@]1(O3)OC [C@H](CC1)C)C)O)C)C [C@@H](CC2)O)C
MOL010395	4′,5,7-trihydroxy-6-methyl-8-methoxy-homoisoflavanone	330.36	89.7	0.33	O1C [C@H](C (=O)C2=C(C(=C(C(=C12)OC)O)C)O)CC1=CC=C(C=C1)O
MOL010396	4′,5,7-trihydroxy-6,8-dimethyl-homoisoflavanone	314.36	59.76	0.3	O1C [C@H](C (=O)C2=C(C(=C(C(=C12)C)O)C)O)CC1=CC=C(C=C1)O
MOL010408	polygosides E_qt	412.67	38.73	0.78	C1 [C@H]2 [C@H](C3=CC=C ([C@]3(C1)C)[C@@H](C (=O)CC [C@@H](CO)C)C)CC=C1 [C@@]2(CC [C@@H](C1)O)C
MOL010411	4′,5,7-trihydroxy-6-methyl-homoisoflavanone	300.33	82.94	0.27	C12=CC(=C(C(=C1C(=O)[C@H](CO2)CC1=CC=C(C=C1)O)O)C)O
MOL010412	4′-methoxy-5,7-dihydroxy-6,8-dimethyl-homoisflavanone	328.39	57.14	0.34	C12=C(C(=C(C(=C1C(=O)[C@H](CO2)CC1=CC=C(C=C1)OC)O)C)O)C
MOL000332	n-coumaroyltyramine	283.35	85.63	0.2	c1c (ccc (c1)/C=C/C (=O)NCCc1ccc (cc1)O)O
MOL000483	(Z)-3-(4-hydroxy-3-methoxy-phenyl)-N-[2-(4-hydroxyphenyl)ethyl]acrylamide	313.38	118.35	0.26	C (=O)(/C=C\C1=CC=C(C(=C1)OC)O)NCCC1=CC=C(C=C1)O

### Construction of the intersection targets PPI network

**FIGURE 4:**
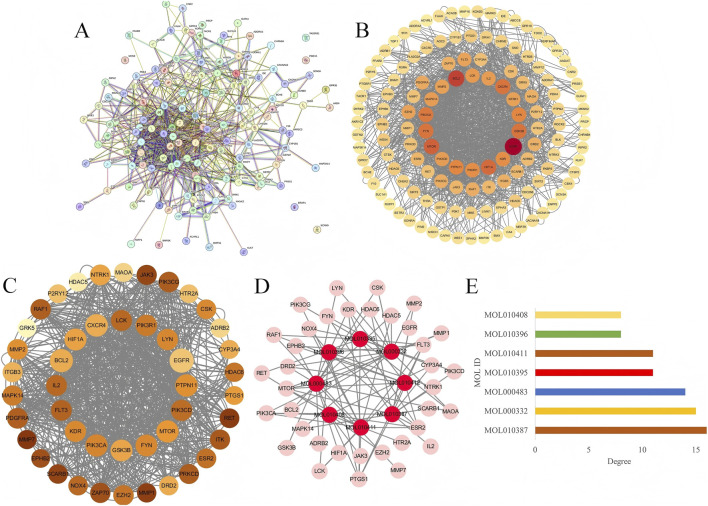
Construction of protein-protein interaction (PPI) network and compound-core target network. **(A)** An initial PPI network of 137 intersection genes. **(B)** PPI network of 132 intersection targets. **(C)** PPI network of 46 core targets **(D)** Compound-core target network between PO active components and IPF-related core targets. **(E)** statistical analysis of PO active components against IPF-related core targets.

To elucidate the interactions among the identified intersection targets, all 137 targets were uploaded to the STRING online platform with a confidence score threshold of ≥0.4 to construct an initial PPI network (Figure 4A). Isolated targets were excluded, resulting in 132 interacting targets (Supplementary Data 5). The remaining targets were further analyzed using Cytoscape 3.8.2 and the CytoNCA plugin to calculate degree values, which were positively correlated with the intensity of target node colors in the network (Figure 4B; Supplementary Data 6). To identify core targets of PO in IPF treatment, 46 targets with a degree value above the average (average degree: 21.36) were selected to construct a core target network (Figure 4C, Supplementary Data 7). Furthermore, an “active component-core target” network was generated by integrating the 8 active components of PO with the 46 core targets in Cytoscape 3.8.2. The resulting network demonstrated that these 46 core targets showed strong associations with the 8 active components (Figure 4D), and statistical analysis confirmed the number of core targets linked to each component (Figure 4E), suggesting that these targets likely represented major regulatory nodes underlying the therapeutic effects of PO against IPF.

### KEGG and GO enrichment analysis

**FIGURE 5:**
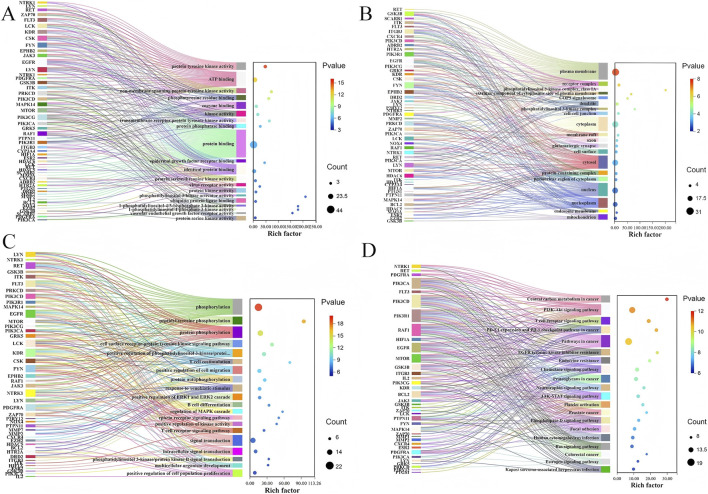
The Top 20 Items of Gene Ontology (GO) and Kyoto Encyclopedia of Genes and Genomes (KEGG) Enrichment Analysis. **(A)** Bubble diagram of molecular function (MF) enrichment items. **(B)** Bubble diagram of cellular component (CC) enrichment. **(C)** Bubble diagram of biological process (BP) enrichment. **(D)** Bubble diagram presenting the top 20 Kyoto Encyclopedia of Genes and Genomes.

To investigate the signaling pathways and biological processes associated with these targets were imported into the DAVID database for KEGG pathway and GO biological process enrichment analysis. The GO enrichment yielded 60 entries for molecular function (MF), 41 entries for cellular component (CC), and 260 entries for biological process (BP) (*p* < 0.05) (Supplementary Data 8). The top 20 high-frequency items for MF, CC, and BP were visualized (Figures 5A–C), indicating that the core targets were primarily involved in GO biological processes related to protein tyrosine kinase activity, plasma membrane, phosphorylation, and so on. Additionally, KEGG pathway enrichment analysis identified 123 pathways (*p* < 0.05) (Supplementary Data 8). Visualization of the top 20 pathways suggested that central carbon metabolism in cancer, the PI3K-Akt signaling pathway, and others (Figure 5D) may represent key mechanisms through which PO’s active components exerted therapeutic effects in alleviating IPF.

### Molecular docking analysis of n-coumaroyltyramine with core targets

**FIGURE 6:**
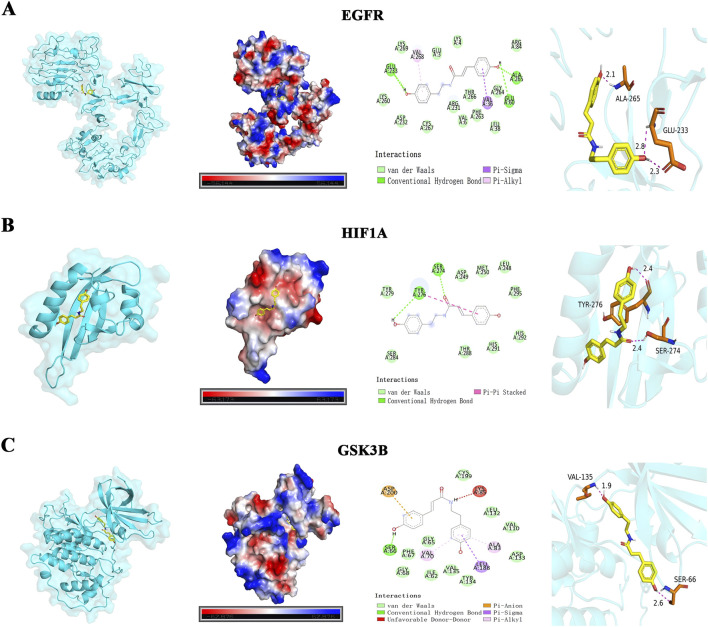
Molecular docking visualization of n-coumaroyltyramine with EGFR, HIF1A, and GSK3B. **(A)** EGFR: Depiction of the three-dimensional binding pose, electrostatic surface representation, 2D interaction diagram, and 3D interaction profile of n-coumaroyltyramine within the EGFR binding pocket. **(B)** HIF1A: Representation of n-coumaroyltyramine bound to the HIF1A active site, including 3D binding conformation, electrostatic surface mapping, 2D interaction schematic, and 3D interaction details. **(C)** GSK3B: Visualization of n-coumaroyltyramine in the GSK3B binding pocket, featuring the 3D binding mode, electrostatic surface view, 2D interaction diagram, and 3D interaction model.

Molecular docking was employed to validate the direct binding of the active components to the core protein targets. The binding energies between the eight active compounds and the top five core targets (degree >68) were evaluated, and all exhibited favorable docking results with binding energies below −6.0 kcal/mol (Table 2). Binding energies below −7.0 kcal/mol are generally regarded as indicative of a high probability of ligand–receptor interaction. Notably, n-coumaroyltyramine demonstrated strong binding affinities toward several key targets, particularly with EGFR (−7.7 kcal/mol), HIF1A (−7.2 kcal/mol), and GSK3B (−7.8 kcal/mol). Consequently, these three targets were selected for further binding mode visualization and analysis. Visualization of the docking results revealed that n-coumaroyltyramine stably occupied the active sites of EGFR, HIF1A, and GSK3B, establishing multiple stabilizing interactions with key amino acid residues. In EGFR, the ligand formed hydrogen bonds and hydrophobic contacts with ALA-265 and GLU-233 (Figure 6A). In HIF1A, hydrogen bonds and π–π stacking interactions were observed with TYR-276 and SER-274 (Figure 6B). For GSK3B, n-coumaroyltyramine established hydrogen bonds and π–anion interactions with residues including VAL-135 and SER-66 (Figure 6C). These interactions suggested a stable and tight binding mode in all three targets. In summary, n-coumaroyltyramine exhibited strong binding affinity and stable interaction patterns with EGFR, HIF1A, and GSK3B, reinforcing its potential role as a key active component in the multi-target and multi-pathway regulatory mechanisms of PO in IPF.

**TABLE 2:** The binding affinity between the core targets and active compounds.

Compounds	Core targets				
​	EGFR	BCL2	MTOR	HIF1A	GSK3B
MOL010412	−7.7	−6.8	−6.4	−7.2	−7.1
MOL010411	−7.6	−6.5	−6.9	−7.2	−7.7
MOL010408	−7.6	−6.5	−6.8	−6.5	−7.7
MOL010396	−7.6	−7.3	−6.8	−7.1	−7.9
MOL010395	−7.6	−6.5	−6.6	−7.3	−7.9
MOL000483	−7.5	−6.9	−6.8	−7.2	−7.6
MOL000332	−7.6	−6.9	−6.8	−7.0	−8.0
MOL010387	−7.5	−6.8	−6.7	−7.1	−8.0

### Molecular dynamics verification of n-coumaroyltyramine–protein binding stability

**FIGURE 7:**
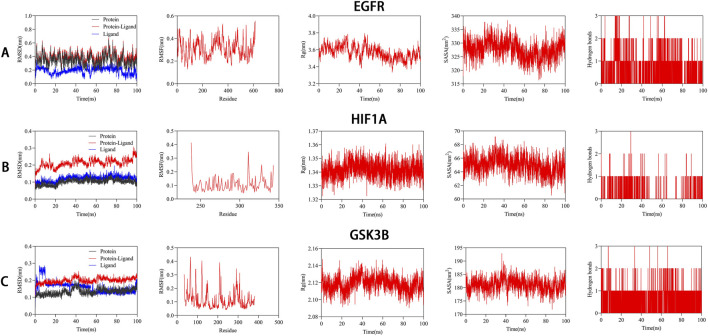
Molecular dynamics simulation of n-coumaroyltyramine bound to EGFR, HIF1A, and GSK3B. **(A–C)** RMSD (Column 1), RMSF (Column 2), Rg (Column 3), SASA (Column 4), and intermolecular hydrogen bond (Column 5) profiles for the EGFR **(A)**, HIF1A **(B)**, and GSK3B **(C)** complexes over 100 ns.

To further evaluate the dynamic stability of n-coumaroyltyramine binding to EGFR, HIF1A, and GSK3B, 100-ns all-atom MD simulations were performed for each complex. The RMSD trajectories (Figure 7A, Column 1) showed that the EGFR complex gradually stabilized within the range of 0.30–0.50 nm, which is acceptable given the intrinsic flexibility of this multi-domain kinase. In comparison, the HIF1A and GSK3B complexes exhibited lower deviations (∼0.20 nm), remaining within the typical range of stable protein–ligand systems (Figures 7B,C, Column 1). These results indicated that ligand binding did not induce major conformational perturbations in any of the proteins. RMSF profiles (Figures 7A–C, Column 2) demonstrated overall low residue fluctuations across the three complexes, with minimal variation in the binding pocket regions, reflecting stable local environments for ligand engagement. Consistent trends were observed in the radius of gyration (Rg) (Figures 7A–C, Column 3) and solvent-accessible surface area (SASA) plots (Figures 7A–C, Column 4), which remained stable throughout the simulations, further supporting preserved global structural compactness and solvent exposure. Intermolecular hydrogen-bond analysis revealed recurrent formation of 1–3 hydrogen bonds between n-coumaroyltyramine and each protein during the 100-ns trajectories, with fluctuating but sustained occupancy patterns (Figures 7A–C, Column 5). These transient yet persistent H-bond interactions, together with stable hydrophobic contacts, supported the formation of robust and energetically favorable binding interfaces. Collectively, the MD analyses confirmed that n-coumaroyltyramine maintained stable interactions with EGFR, HIF1A, and GSK3B under dynamic conditions, reinforcing its potential as a multi-target ligand with favorable binding stability.

### Effect of n-coumaroyltyramine on BEAS-2B cell viability

**FIGURE 8:**
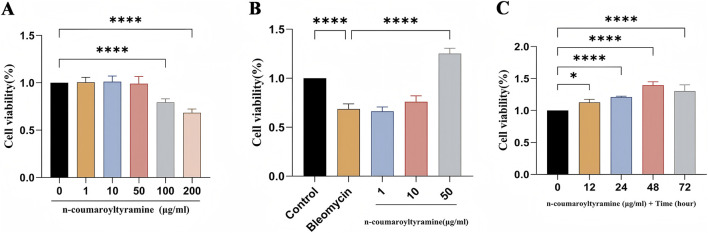
Effects of N-coumaroyltyramine on BEAS-2B cell viability. **(A)** Dose–response analysis (0–200 μg/mL, 48 h) identified 50 μg/mL N-coumaroyltyramine as a safe concentration with minimal cytotoxicity. **(B)** In a bleomycin-induced injury model (10 μg/mL, 48 h), treatment with 50 μg/mL N-coumaroyltyramine significantly improved cell viability. **(C)** A time-course assay (0–72 h) at 50 μg/mL showed maximal protective effects at 48 h.

N-Coumaroyltyramine, one of the main active components of PO, exhibited favorable binding interactions with EGFR, HIF1A, and GSK3B. Based on these findings, the present study further evaluated the potential therapeutic effects of n-Coumaroyltyramine in IPF. The CCK-8 assay was used to assess the effects of n-coumaroyltyramine on BEAS-2B cell viability. BEAS-2B cell viability was first assessed by CCK-8 to characterize the dose–toxicity profile of n-coumaroyltyramine. A concentration–response screen (0, 1, 10, 50, 100, and 200 μg/mL for 48 h) demonstrated a clear cytotoxicity threshold, and 50 μg/mL was identified as a safe concentration with minimal viability loss (Figure 8A). This concentration was subsequently applied to a 10 μg/mL bleomycin-induced injury model to evaluate pharmacological efficacy. Compared with the bleomycin group, N-coumaroyltyramine at 50 μg/mL significantly restored BEAS-2B cell viability, indicating a protective effect (Figure 8B). After establishing the optimal concentration, a time-course assay (0, 12, 24, 48, and 72 h at 50 μg/mL) showed a time-dependent enhancement of efficacy, with maximal improvement at 48 h (Figure 8C). Collectively, these data demonstrated that n-coumaroyltyramine exhibited acceptable cytotoxicity at 50 μg/mL, exerted protective effects against bleomycin-induced cellular injury, and achieved optimal activity following 48 h of exposure.

### Evaluation of n-coumaroyltyramine’s protective effects on bleomycin-induced cellular damage in BEAS-2B cells

**FIGURE 9:**
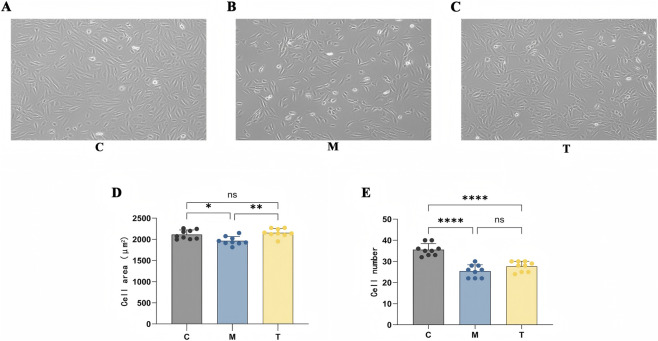
Morphological changes in BEAS-2B cells treated with bleomycin + n-coumaroyltyramine. **(A)** Control group: Normal epithelial morphology with flat, polygonal cells and close cell-to-cell contact. **(B)** Bleomycin-treated group: Cells showed shrinkage, rounding, vacuolization, and increased intercellular gaps. **(C)** Bleomycin + n-coumaroyltyramine-treated group: Improved morphology with reduced shrinkage and vacuolization, restoring epithelial-like appearance. **(D)** Cell area quantification: Statistical analysis of cell surface area in each treatment group. **(E)** Cell count quantification: Quantitative comparison of cell numbers across groups. Data were presented as mean ± SD (n ≥ 3). *p < 0.05, **p < 0.01, ***p < 0.001 vs. Control group; #*p* < 0.05, ##p < 0.01 vs. Bleomycin-treated group. All key parameters were quantified using three independent biological replicates (n = 3), with three randomly selected fields analyzed per replicate. All images were acquired at ×100 magnification. Abbreviations: C, Control group; M, Bleomycin-treated group; T, Bleomycin + n-coumaroyltyramine-treated group.

To evaluate the therapeutic potential of n-coumaroyltyramine in IPF, a cellular injury model was established by exposing BEAS-2B cells to 10 μg/mL bleomycin for 48 h. Cells were then treated with 50 μg/mL n-coumaroyltyramine for 48 h, and morphological changes were assessed. In the control group, BEAS-2B cells exhibited typical epithelial morphology, characterized by a flat, polygonal shape and tight cell–cell contact (Figure 9A). Bleomycin treatment induced pronounced morphological alterations, including cell shrinkage, rounding, increased intercellular gaps, cytoplasmic vacuolization, and reduced cell density, consistent with fibrosis and cellular damage (Figure 9B). Treatment with n-coumaroyltyramine markedly ameliorated these abnormalities, restoring epithelial-like appearance, reducing shrinkage and vacuolization, and decreasing intercellular separation (Figure 9C). Consistent with these findings, quantitative analysis showed that bleomycin markedly reduced cell area (*p* < 0.001), whereas co-treatment with n-coumaroyltyramine significantly restored it (*p* < 0.01) to a level comparable to the control (*p* > 0.05) (Figure 9D). Likewise, bleomycin significantly decreased cell number (*p* < 0.001), while n-coumaroyltyramine alleviated this reduction, resulting in a significantly higher cell count than the bleomycin group (*p* < 0.01) and similar to control levels (*p* > 0.05) (Figure 9E). These data indicated that n-coumaroyltyramine effectively mitigated bleomycin-induced morphological and quantitative damage in BEAS-2B cells.

### Regulatory effects of n-coumaroyltyramine on targets in an IPF cell model

**FIGURE 10:**
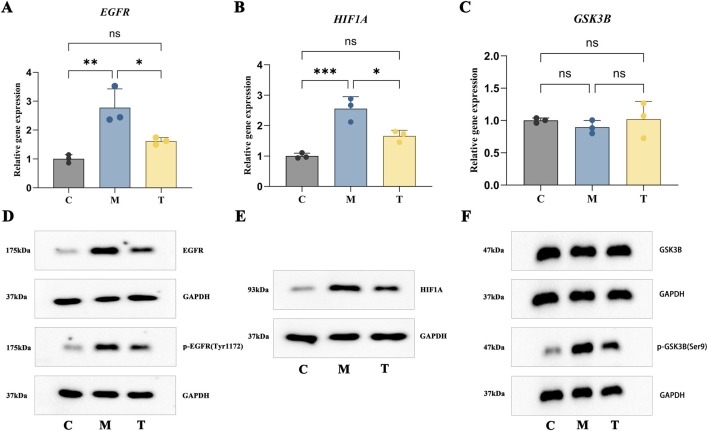
Regulation of fibrosis-related targets by n-coumaroyltyramine in BEAS-2B cells. **(A–C)** qPCR analysis: *EGFR* and *HIF1A* mRNA levels were upregulated by bleomycin but downregulated after n-coumaroyltyramine treatment; *GSK3B* mRNA levels remained unchanged. **(D–F)** Western blot analysis: n-coumaroyltyramine treatment inhibited the expression of EGFR, p-EGFR, HIF1A, GSK3B and p-GSK3B proteins compared to the bleomycin-treated group. Data are presented as mean ± SD; *p < 0.05; **p < 0.01; ns, not significant. C: Control group; M: bleomycin-treated group; T: bleomycin + n-coumaroyltyramine-treated group.

To investigate the therapeutic potential of n-coumaroyltyramine in IPF, fibrosisrelated targets were examined by qPCR and Western blot WB analyses. The qPCR results showed that mRNA expression levels of *EGFR* and *HIF1A* were significantly upregulated in the bleomycin-treated group compared with the control, consistent with their pro-fibrotic roles. N-coumaroyltyramine treatment subsequently reduced their expression (Figures 10A,B). In contrast, no significant changes in GSK3B mRNA expression were observed between the bleomycin-treated and ncoumaroyltyramine-treated groups (Figure 10C). Western blot analysis furthermore that n-coumaroyltyramine inhibited the expression of EGFR, p-EGFR, HIF1A, and p-GSK3B (Figures 10D-F). Collectively, these data suggested that ncoumaroyltyramine modulated key fibrosis-related targets, such as EGFR, HIF1A, and GSK3B to attenuate bleomycin-induced fibrosis in BEAS-2B cells.

## Discussion

IPF, an irreversible disorder with a median survival of only 3–5 years, increasingly represented a major global health burden (Selvarajah et al., 2023). Elucidating the pathogenic mechanisms of IPF, particularly the identification of key targets linked to disease onset and progression, enabled early clinical diagnosis and facilitated therapeutic development. Targeting pivotal regulatory molecules could also accelerate the discovery of effective therapeutic strategies. In this context, WGCNA provided an innovative systems-biology framework for identifying hub genes closely associated with disease progression (Zhang and Horvath, 2005). In parallel, network pharmacology, characterized by high-throughput and integrative analytical capabilities, offered an effective strategy for evaluating the therapeutic potential of multicomponent traditional Chinese medicines (Zhang et al., 2023b). In the present study, WGCNA was applied to identify gene modules associated with IPF, subsequent PPI networks construction and enrichment analyses allowed the selection of corresponding hub genes. Network pharmacology was further employed to assess the regulatory relevance of bioactive components of PO to these IPF-related targets, thereby preliminarily elucidating potential therapeutic mechanisms.

This study identified a total of 46 core therapeutic targets involved in the treatment effect of PO against IPF. Among these, EGFR, HIF1A, and GSK3B emerged as dominant regulators of the pathological state of IPF. EGFR, a prototypical member of the ErbB tyrosine kinase receptor family, played essential role in normal cellular growth and differentiation (Cohen et al., 1980; Ullrich et al., 1984). Mechanistic studies in pancreatic cancer showed that that the EGFR/ERBB2 signaling pathway could be activated by transforming growth factor-β (TGF-β), thereby accelerating PDAC metastasis (Mucciolo et al., 2024). Although EGFR activation did not directly induce myofibroblast transformation in fibrotic kidneys, it promoted fibroblast migration and proliferation. More importantly, specific EGFR inhibition could significantly improve the renal interstitial fibrosis (Cao et al., 2023). HIF-1, a helix-loop-helix transcription factor, was involved in various biological processes including angiogenesis, cell proliferation, and glucose metabolism (Jiang et al., 1997). Under extreme hypoxic conditions, heterodimeric HIF1A-HIF1B could stabilize and negatively regulate RIPK3-dependent necroptosis, thus alleviating arthritis pathology (Lyu et al., 2024). Additionally, HIF1A also dynamically regulated mitochondria ROS levels under hypoxia, preventing fibroblast proliferation and ultimately delaying cardiac fibrosis progression (Janbandhu et al., 2022). GSK3, a multifunctional serine/threonine protein kinase, comprised two isoforms, namely, GSK3A and GSK3B (Sharma et al., 2020). GSK3B induced axonal autophagy via phosphorylation of the BCL-2 family protein MCL1, which represented a critical mechanism underlying Wallerian degeneration (Wakatsuki et al., 2017). Moreover, elevated follicle-stimulating hormone levels directly inactivated GSK-3β and exacerbated renal tubular interstitial fibrosis, indicating a close association with renal fibrosis fibrogenesis (Zhang et al., 2019).

Among the top 20 enriched pathways identified in this study, the PI3K-AKT signaling pathway, a central regulator of cell survival, proliferation, and metabolism, had been widely reported to be aberrantly activated in PF (Wang et al., 2022a). The present findings suggested that n-coumaroyltyramine exerted anti-fibrotic effects through modulation of this axis. EGFR activation triggered the ERK1/2 pathway, which mediated phosphorylation-dependent inactivation of GSK3B. Inactivated GSK3B failed to degrade β-catenin, thereby promoting its nuclear translocation and transcriptional activation of profibrotic genes through TCF/LEF (Mo et al., 2022). As a core effector of the Wnt/β-catenin signaling pathway, aberrant activation and nuclear translocation of β-catenin in fibroblasts directly elevated the expression of fibrosis-related genes, including collagen and α-smooth muscle actin (α-SMA), facilitated fibroblast activation and proliferation, and ultimately driving the progression of pulmonary fibrosis (Hu et al., 2022). Additional evidence demonstrated that blocking EGFR phosphorylation dependent PI3K/Akt activation significantly ameliorated hepatic steatosis, oxidative stress, and ferroptosis in a metabolic dysfunction-associated steatotic liver disease (Wang et al., 2024). Likewise, the traditional Chinese medicine compound Shenfu injection attenuated heart failure-associated myocardial fibrosis by suppressing the PI3K/AKT/HIF1A signaling (Li et al., 2025). These findings collectively indicated the notion that PI3K/Akt served as a central regulator of fibrosis in multiple tissue types.

## Conclusion

This study integrated WGCNA -based transcriptomic analysis with network pharmacology prediction to elucidate the multi-component, multi-target mechanisms by which PO intervened in IPF. WGCNA identified five gene modules associated with IPF comprising 3,541 genes, while TCMSP screening identified eight active PO constituents. Subsequent PPI interactions, enrichment analysis, and molecular docking results indicated that representative active compounds (MOL010412, MOL010387, MOL010395, MOL010396, and MOL000332) directly interacted with core targets (EGFR, HIF1A, and GSK3B). Experimental validation further demonstrated that n-coumaroyltyramine alleviated bleomycin-induced fibrosis in BEAS-2B cells by modulating EGFR, GSK-3β, and HIF-1α-related pathways. Together, these findings provided modern pharmacological support for the traditional application of PO in IPF and identified high-value compounds and actionable therapeutic targets. However, given the chemical complexity of PO, this study only addressed selected components, and potential additive, synergistic, or antagonistic interactions remained uncharacterized. In addition, although the initial network-based predictions were supported by cellular experiments, the proposed multi-target regulatory mechanisms require further validation in more comprehensive biological systems, including advanced *in vitro* models and *in vivo* studies. Moreover, the therapeutic relevance of PO should be evaluated in comparison with standard IPF treatments, such as pirfenidone and nintedanib, in well-established animal models to determine whether PO provides comparable or complementary pharmacological benefits. Future investigations should therefore prioritize multi-component pharmacology, *in vivo* mechanistic studies, and translational evaluation in clinical cohorts to establish the therapeutic potential of PO in IPF.

## Data Availability

The datasets presented in this study can be found in online repositories. The names of the repository/repositories and accession number(s) can be found in the article/Supplementary Material.
