# Supplementary figures and images for: Pharmacological mechanism of active components in Polygonatum odoratum for idiopathic pulmonary fibrosis: a study integrating bioinformatics and experimental validation
Source: Front Pharmacol. 2026 Mar 2;17:1717994. doi: 10.3389/fphar.2026.1717994 (PMC12989546; doi:10.3389/fphar.2026.1717994)

Module Preservation Median Rank

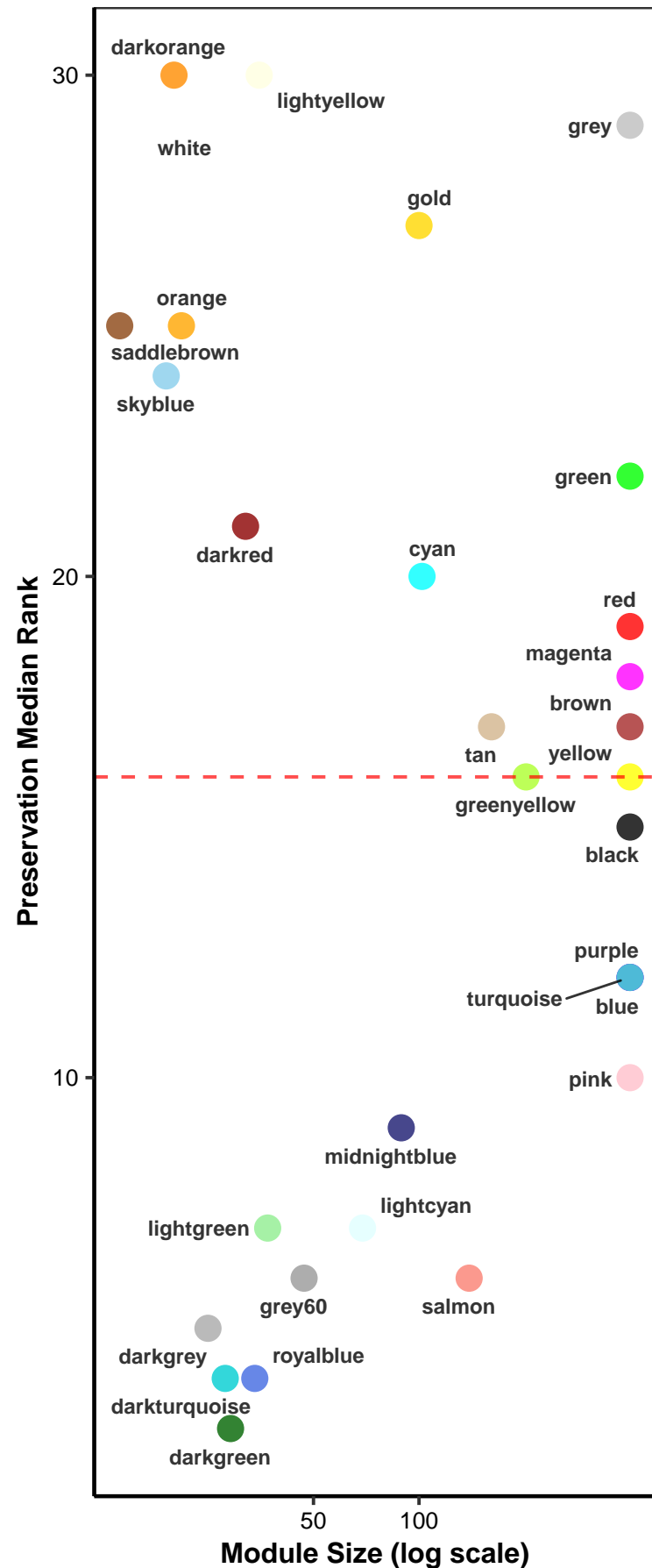

Module Preservation Zsummary

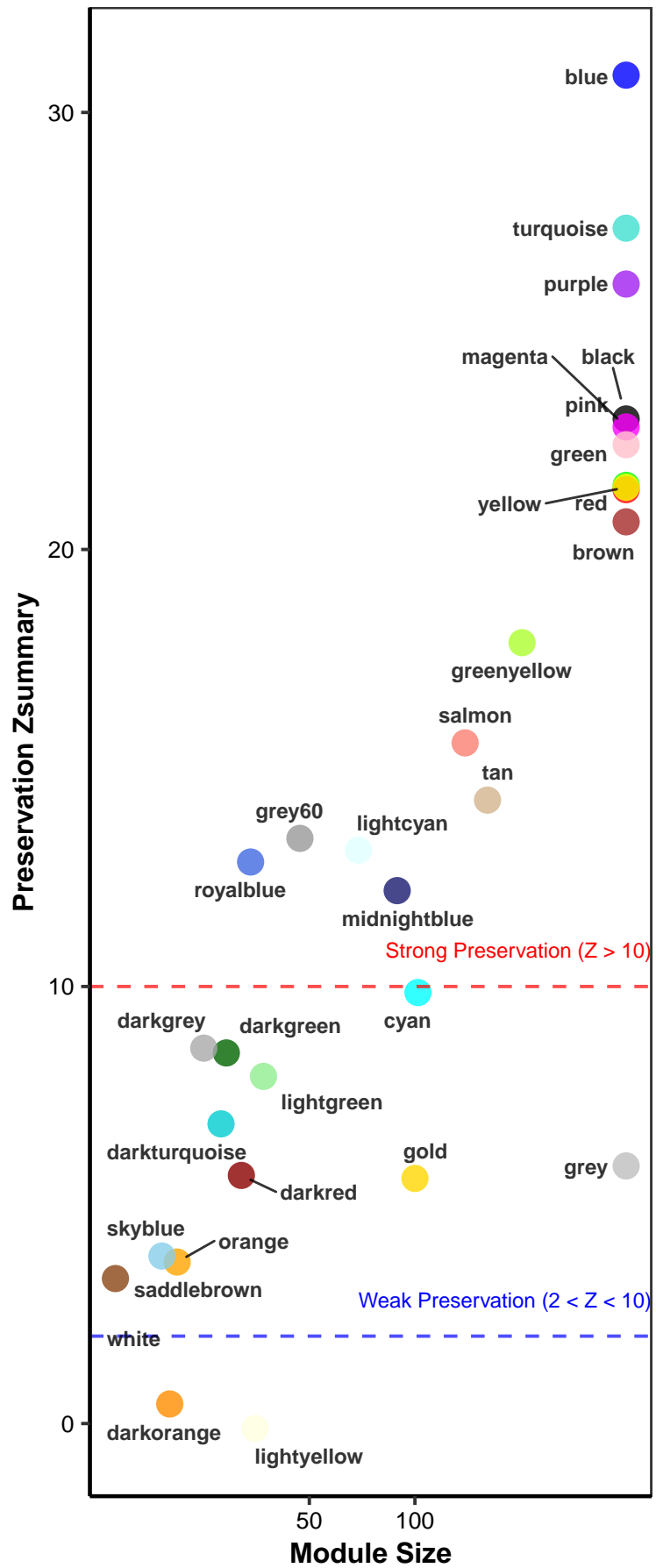

Supplement: Supplementary file 2 [file DataSheet1.pdf]
